# Quasi-static force requirements are not sufficient to explain arolium engagement in climbing Argentine ants

**DOI:** 10.1242/jeb.253096

**Published:** 2026-08-26

**Authors:** Yakun Cao, Andrew Chacon, Agasthya Valluri, Liam O'Connor Mueller, Nick Gravish

**Affiliations:** ^1^ Department of Mechanical and Aerospace Engineering, University of California San Diego, La Jolla, CA 92093, USA; ^2^ Department of Molecular Biology, University of California San Diego, La Jolla, CA 92093, USA

**Keywords:** Locomotion, Foot adhesion, *Linepithema humile*, Tripod gait, Acceleration

## Abstract

Argentine ants (*Linepithema humile*) utilize adhesive pads (arolia) to climb smooth surfaces. Previous research found that ants can adjust their individual arolium engagement according to their locomotion mode. However, it remains unclear how they distribute arolium engagement across multiple limbs to climb effectively, and how arolium engagement varies within a climbing step. As the arolium is a well-known adhesive organ, we hypothesized that engagement across different legs is distributed according to the normal forces required for balancing the body during climbing. To test this, we measured Argentine ants' arolium engagement on a vertical glass surface using a frustrated total internal reflection (FTIR) sensor and compared it with the required normal forces from a quasi-static model. Contrary to the required normal force, the measured arolium engagement was asymmetric between upward and downward climbing, and changed over time. Our results indicate that quasi-static force requirements are not sufficient to explain arolium engagement in climbing Argentine ants, and suggest that other factors, such as body dynamics, ant anatomy and behavioral preferences, should be included.

## Teaser

**Summary:** Foot forces required for static balance do not fully explain how climbing ants use their adhesive pads across legs; other factors, such as body acceleration during each step, also matter.

## INTRODUCTION

Some ant species, such as Argentine ants (*Linepithema humile*), can navigate smooth artificial surfaces such as glass windows and metal tables to forage (Mallis, 1942; Choe et al., 2012; Reid et al., 2012). The key anatomical feature that enables this ability is the arolium (Endlein and Federle, 2008; Brechka, 2024), a specialized adhesive pad located on the 5th tarsomere of ants' legs between the two claws (Federle et al., 2001; Gorb, 2007). To understand how ants can navigate on smooth substrates, it is important to explore how ants use their arolia.

Arolia can help ants to generate adhesion forces more than 40 times their body weight (Federle et al., 2000). However, ants do not always engage their arolia (Endlein and Federle, 2015) and often avoid full engagement (Federle and Endlein, 2004), likely because the increased engagement can hinder rapid disengagement. This is supported by previous findings that when exposed to strong detachment forces such as carrying weights while walking upside down (Federle and Endlein, 2004), resisting puffs of air or holding on to a rotating drum (Federle et al., 2000), ants walked very slowly or stayed motionless. Given the impact of full arolium engagement on ant mobility, to perform movements efficiently, they may carefully adjust the adhesive contact of their arolia to achieve a balance between generating sufficient adhesion force and maintaining locomotor speed.

Previous studies provided evidence that ants can adjust arolium engagement (Federle et al., 2001) and disengagement (Federle et al., 2002), as well as the adhesive contact area (Federle and Endlein, 2004) according to their locomotion mode. When weaver ants walked upright, their 5th tarsomeres were mostly lifted with the arolia disengaged, while their arolia would adhere to the top surfaces when walking upside down (Endlein and Federle, 2015). Moreover, when a load was added to the upside-down ants, the contact area of their arolia increased (Federle and Endlein, 2004).

These prior findings were obtained at the level of a single arolium (Federle et al., 2001; Endlein and Federle, 2015; Federle and Endlein, 2004). However, climbing often involves multiple feet in contact with the surface at the same time. Therefore, to understand how Argentine ants achieve efficient climbing on smooth substrates, it is important to determine how they use their arolia across different legs. Previous work has shown that arolium engagement is typically present when a leg generates adhesion force (Endlein and Federle, 2015). Thus, we hypothesized that Argentine ants distribute their arolium engagement on different legs according to the force requirement in the normal direction. Specifically, we expected Argentine ants to engage their arolia on legs required to generate adhesion forces, and to disengage the arolia on legs required to generate compression forces. Furthermore, the leg with the higher required force should exhibit more intense arolium engagement.

To test our hypothesis, we first needed to determine the required normal forces on stance-phase limbs. Previous research demonstrated that the normal force pattern of geckos – adhesion forces generated by the legs above the center of mass – could be explained by a quasi-static sagittal-plane force model (Autumn et al., 2006; Wang et al., 2011). Additionally, similar normal force patterns have been observed in weaver ants (Endlein and Federle, 2015). These findings suggest that a quasi-static force model can be a simple but reasonable choice. Additionally, if arolium usage is impacted by body dynamics, the quasi-static model could serve as a null model to compare with. As ants often climb with three feet touching the ground (Humeau et al., 2019; Seidl and Wehner, 2008), we proposed a 3D tripod quasi-static force model to predict the normal forces.

We then tested our main hypothesis – whether Argentine ants distribute arolium engagement according to normal force requirements – by checking whether the measured engagement on a glass surface during vertical climbing matched the normal force pattern predicted by the quasi-static model. Using a frustrated total internal reflection (FTIR) sensor, we detected which arolia on the stance-phase legs were engaged with the glass according to the occurrence of illumination and measured the light flux from each illuminated contact as an indicator of engagement intensity. We performed three specific analyses on Argentine ant upward and downward climbing: (1) we checked whether the arolia required to generate adhesion forces were engaged; (2) we examined whether the ratio of arolium engagement intensity between different legs was positively associated with the ratio of their model-predicted required adhesion forces; and (3) given the time invariance of quasi-static force requirements, we tested whether the engagement status of each arolium stayed the same during each step.

## MATERIALS AND METHODS

### Study animals

Argentine ants, *Linepithema humile* (Mayr 1868), with surrounding soil were collected from eight locations across the University of California San Diego campus, La Jolla, CA, USA, between 20 June and 25 July 2023 (Fig. S1). We refer to the ants collected from each location as a separate ‘collection’. The collected ants were kept in a temperature-regulated laboratory room and were fed with ant nectar (byFormica Sunburst Ant Nectar) and water *ad libitum.*

### Experimental apparatus and procedure

**Fig. 1.**
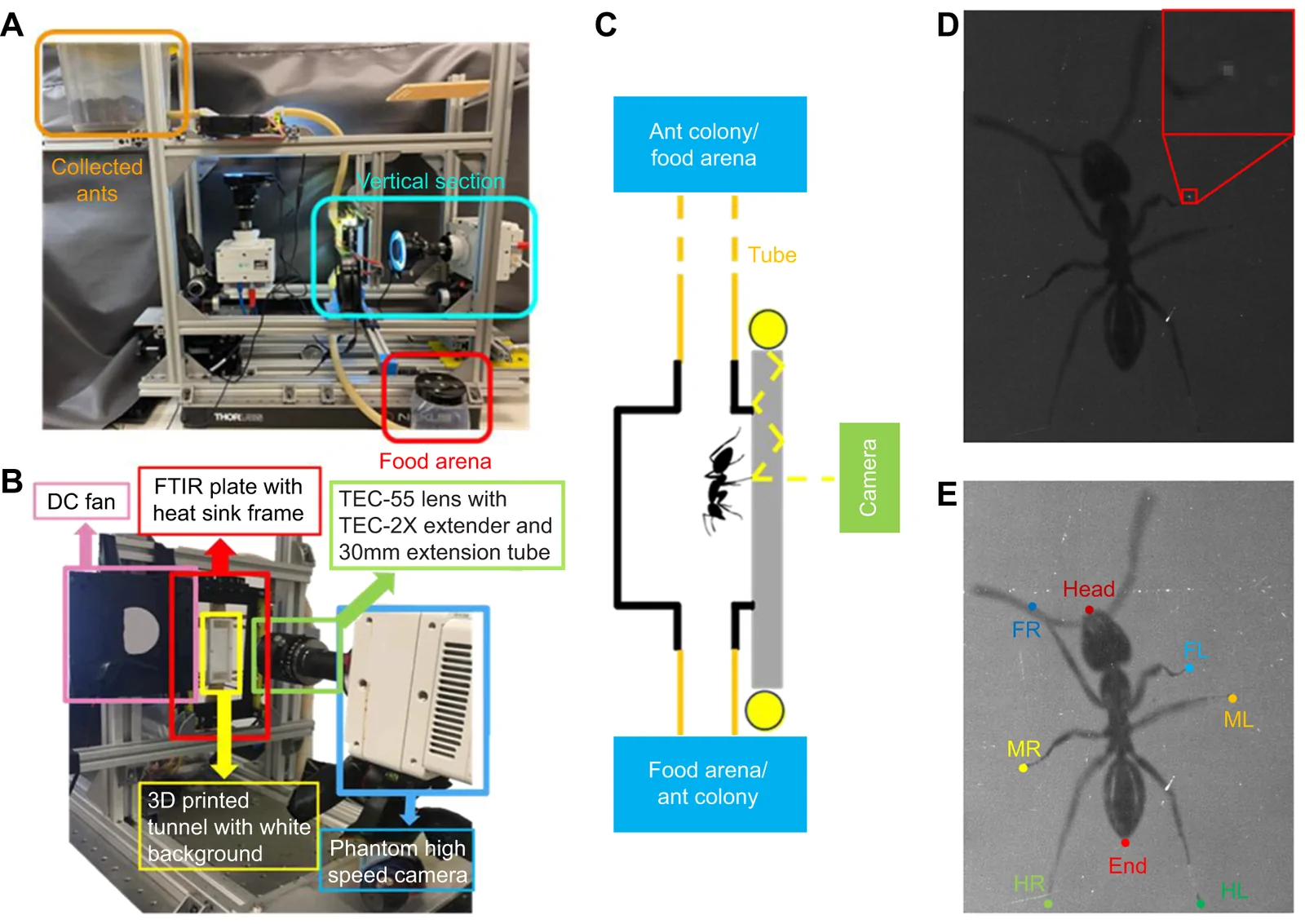
**Experimental set-up.** (A) Overall experimental set-up, showing the plastic containers for the collected ants and food arena, and the vertical section. (B) Detailed set-up for the vertical section, showing the vertically mounted frustrated total internal reflection (FTIR) plate with a heat sink frame, a Phantom high-speed camera (with a TEC-55 lens, a TEC-2X lens extender and a 30 mm extension tube) and a 3D printed white bottomless tunnel. (C) Diagram of the experiment. (D) Raw image of an ant during upward climbing. In this figure, the front left (FL) and middle right (MR) feet tips were illuminated, indicating the engagement of arolia on these two legs. The inset shows the illuminated FL arolium engagement (boxed region) on a magnified scale. (E) Ant image with body parts labeled using DeepLabCut. The image shown is a contrast-adjusted version of D, with contrast modified solely for visualization purposes. For tarsal tip labels, F, M and H indicate front, middle hind foot, respectively; L and R indicate left and right.

A custom-built experimental apparatus was used to record the body kinematics and arolium engagement in Argentine ants (Fig. 1A). The apparatus consisted of a vertically mounted FTIR sensor, a 3D printed white bottomless tunnel (PLA), a Phantom VEO 410 L high-speed camera (Vision Research, Wayne, NJ, USA) and two plastic containers (Fig. 1A,B).

The FTIR sensor is a borosilicate glass plate (152.4 mm×101.6 mm×9.5 mm; McMaster-Carr, Elmhurst, IL, USA) surrounded by a 13 mm wide 24 V LED light strip (6000±300 K, Daylight White; JOYLIT, Shenzhen, China). When light travels through the glass plate, light rays that are at an angle below the angle of refraction with respect to the plate surface will be reflected internally, as the glass plate has a higher index of reflection than its surrounding air. However, when an object such as the ant's arolium is in nanoscale proximity to the outer plate surface, light can be transmitted through the surface (Fig. 1C). The transmitted light from close contact points is then scattered out of the glass plate and toward a light-sensitive camera, where it is recorded as an illuminated spot (Fig. 1D). This technology has been used previously to analyze animal foot contact (Gravish et al., 2010; Mendes et al., 2013; Wang et al., 2021). A DC fan and a heat sink frame were used around the FTIR sensor to maintain a reasonable temperature range for ants to walk on. The Phantom high-speed camera is equipped with a 55 mm Computar telecentric lens (IVS Imaging, Coppell, TX, USA), a TEC-2X extender (IVS Imaging) and a 30 mm extension tube.

During the experiment, ants could freely climb on the FTIR sensor (the temperature in the ant climbing area was controlled in the range 22–34°C; Holway et al., 2002; Human et al., 1998; Fig. S2). The high-speed camera was set in image based auto-trigger (IBAT) mode with 400 frames s^−1^ recording frequency. Once ants entered the field of view, the camera automatically captured videos, with each video approximately 618 frames long. The recording session for each collection lasted a minimum of 24 h.

### Step selection

The open-source toolbox DeepLabCut (version 2.3.5) was used to track the six tarsal tips of the feet, the anterior end of the head (‘Head’) and the posterior end of the gaster (‘End’) for each frame (Fig. 1E). The center of body (CoB) was the middle point between the ‘Head’ and ‘End’. All videos were separated into steps (from touch-down to lift-off for a hindlimb; see Supplementary Materials and Methods, ‘Method S1. Step separation’, for details on how steps were separated). For each step, the tripod coordination strength (TCS) was calculated as the ratio between the number of frames in which all three legs of a given tripod were simultaneously in swing phase and the number of frames in which at least one of these legs was in swing phase (Wosnitza et al., 2013). Steps with TCS>0.5 and orientated vertically (±20 deg from the gravity direction) were selected for further analyses. Additionally, within each step, only frames where the ant was in an alternating tripod gait were included in the further analysis. The normalized time for each frame was calculated according to its position in the whole step frame sequences, with 0 and 1 indicating the beginning and end of a step, respectively. Each frame was treated as an observation.

### Arolium engagement calculation

We identified arolium engagement by comparing pixel brightness at the foot with respect to the surrounding background noise (see Supplementary Materials and Methods, ‘Method S2. Calculation of arolium engagement intensity’ for a complete description). Specifically, we used two metrics: (1) a binary representation of whether arolia are engaged/not engaged, which is computed by thresholding the arolium light intensity, and (2) a measure of arolium engagement intensity, which is computed by summing all pixels that exceed the noise threshold.

We verified that the illuminated contacts originated specifically from the arolium and not from other leg structures using a supplementary experiment (see Supplementary Materials and Methods, ‘Method S3. FTIR sensor validation’, and Fig. S3). Because the fine contact details of the arolium are below our optical resolution, we used the integrated FTIR light flux (summed pixel values) from each illuminated contact as a relative measure of total contact area, which we defined as engagement intensity. This interpretation is based on the physical principle of frustrated total internal reflection: a larger area of optical contact scatters more light, resulting in a higher total pixel intensity.

### 3D tripod quasi-static force model

A quasi-static tripod model of climbing was constructed as a point mass with three weightless limbs representing the ant's center of mass (CoM) connected with the three stance phase limbs. The CoM is located a distance *h* from the surface in the *z* direction, and the three stance phase limbs contact the climbing surface. The mass point is subjected to gravity (*m**g***), and 3-dimensional (3D) foot contact forces (**F**_1_, **F**_2_, **F**_3_) act on the tip of all stance-phase limbs (Fig. S4). To maintain the quasi-static force balance, the sum of gravitational (*m**g***) and foot contact forces (**F**_1_, **F**_2_, **F**_3_) must be zero in all three directions, and the sum of torques about the CoM due to the foot contact forces (**τ**_1_, **τ**_2_, **τ**_3_) must be zero for all three axes. These requirements allow us to get the analytical solution for the normal components of the foot contact forces (*F*_1*z*_, *F*_2*z*_, *F*_3*z*_; see Supplementary Materials and Methods, ‘Method S4. 3D tripod quasi-static force model’, for details):

(1) $$F_{1z}=\frac{mgh(y_{3}-y_{2})}{D},$$

(2) $$F_{2z}=\frac{mgh(y_{1}-y_{3})}{D},$$

(3) $$F_{3z}=\frac{mgh(y_{2}-y_{1})}{D},$$

(4) $$D=(x_{1}-x_{3})(y_{2}-y_{3})-(x_{2}-x_{3})(y_{1}-y_{3}),$$

where *F*_1*z*_, *F*_2*z*_, *F*_3*z*_ represent the signed scalar normal components of the foot contact forces, *mg* represents the magnitude of the gravitational force acting on the object, *h* is a positive scalar representing the distance between the point mass and the contacting surface, and *x*_1_, *x*_2_, *x*_3_ and *y*_1_, *y*_2_, *y*_3_ correspond to the *x* and *y* signed scalar components of the foot contact points relative to the CoM. When calculating the required normal force, the measured CoB position was used as an estimate of the CoM position, and the distance ℎ was assumed to remain constant during each step. The potential influence of the variation in *h* on the force predictions is assessed in the Discussion. As the locations of the contacting feet do not change relative to each other during each stance phase, the time dependencies of the foot positions (*x_i_*, *y_i_*) caused by the movement of the CoM compensate in Eqns 1–3. This leads to step-wise time-invariant normal forces, where positive values indicate compression forces and negative values indicate adhesion forces.

### Acceleration calculation

The instantaneous speed at each frame was determined by first calculating the displacement between the CoB positions at the previous and next frames. Then, the component of this displacement that aligned with the fore–aft body axis was taken, and divided by twice the frame duration (central difference method). Acceleration was obtained by applying the same central difference method to the previously computed instantaneous speed. The maximum magnitude of acceleration for each step was defined as the largest absolute value of all frame-wise accelerations.

### Statistical analysis

All statistical analyses were performed in R (v.4.2.3). Because of repeated measurements from different collections of ants from distinct locations and large differences in sample size across collections, we treated each collection as our experimental unit (*k* represents the number of independent collections) and used a two-stage method for all statistical analyses (Gerow et al., 2021). The two-stage method proceeded as follows: (1) a separate binomial (linear) regression model was fitted to each collection to obtain the estimate and its standard error per collection; (2) the collection-specific results were synthesized into an overall result using random-effects meta-analyses (metafor package; Viechtbauer, 2010).

In the first stage, binomial regression models were used to obtain the estimates and standard errors for the arolium engagement probability across foot positions, stance types and normalized time. Linear regression models were used to estimate the relationship between the log-transformed engagement intensity ratio and the log-transformed adhesion force ratio, and the temporal variation of acceleration. Second-order polynomial regression models were used to estimate the quadratic coefficients (*a*) and the linear coefficients (*b*) for the temporal variation of engagement intensity. Vertices (peaks) were calculated as –*b*/2*a*. Standard errors for vertices were obtained using the delta method.

In the second stage, univariate meta-analyses were performed to obtain overall estimates and to test our hypotheses. To test whether outcomes varied with direction (up versus down) or stance type (narrow versus wide), we calculated the corresponding collection-specific differences and synthesized each set of differences using meta-analysis. In our study, we chose 0.05 as the level for statistical significance (α). To account for multiple testing, we applied Holm–Bonferroni correction (Holm, 1979) to *P*-values from tests of whether (1) middle arolium engagement probability varied with direction or stance type, (2) engagement probability varied over time and (3) engagement intensity showed an asymmetric temporal pattern.

## RESULTS

### Stance type and arolium engagement probability

**Fig. 2.**
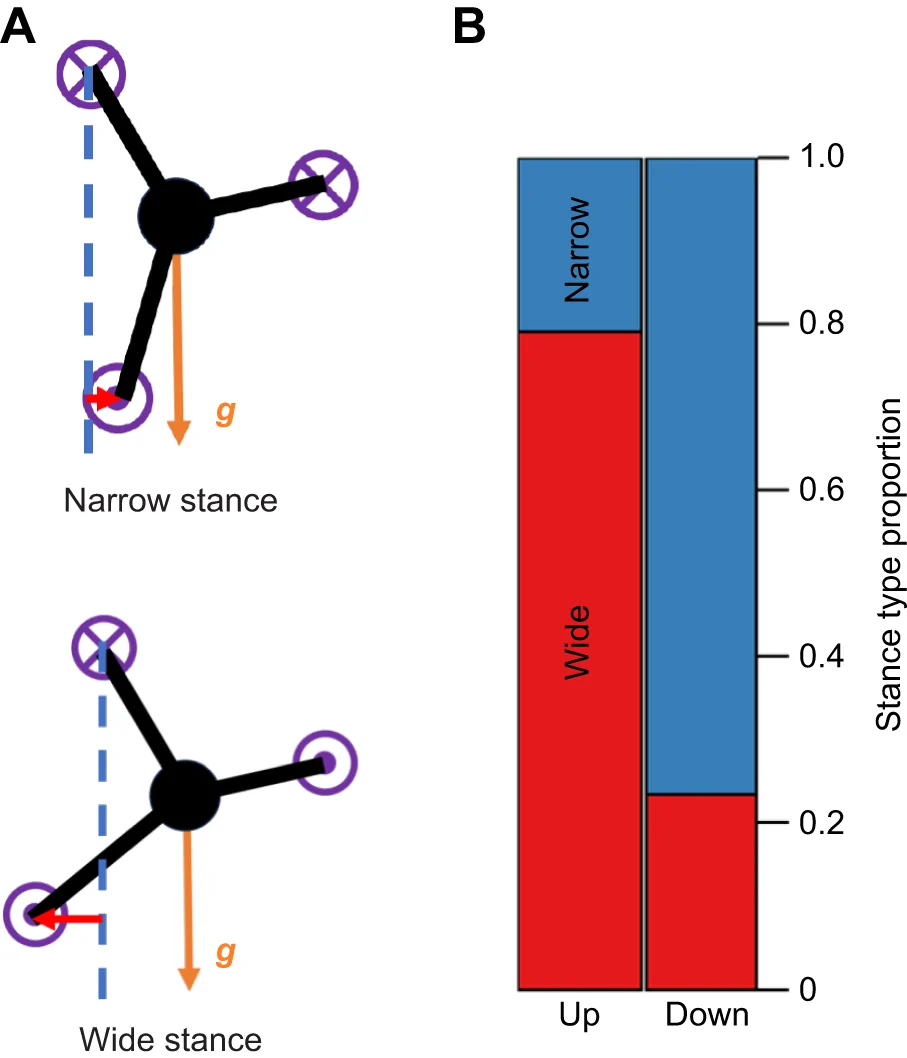
**Stance types and model-predicted normal forces.** (A) Diagram of narrow stance and wide stance. The tripod models in the dorsal plane are shown in black. Gravity (***g***) is represented by the orange arrows. Blue dashed lines and red arrows show the relative lateral position between the uppermost foot and the lowest foot. The purple symbols indicate the required normal force direction for each foot according to the tripod force model: ⊗ denotes an adhesion force, while ⊙ denotes a compression force. (B) Stance type proportion in upward and downward climbing. Red bars represent the proportion of a wide stance, while blue bars show the proportion of a narrow stance. *n*=20,314 observations for upward climbing and *n*=18,093 observations for downward climbing.

We first checked whether the feet that are required to generate adhesion forces exhibited arolium engagement (as indicated by FTIR illumination). Stance limbs were classified as uppermost, middle, or lowest by their vertical position along the gravity axis. According to the quasi-static model, the uppermost foot should always generate adhesion force, while the lowest foot should always generate compression force, regardless of climbing direction. The required normal force direction for the middle foot is also direction independent but depends on whether an ant is in wide stance or narrow stance (Fig. 2A). Narrow stance is when the lowest foot is laterally closer to the CoB than the uppermost foot. Wide stance is when the lowest foot is laterally further from the CoB than the uppermost foot. If an ant is in a narrow stance, their middle leg would be required to generate adhesion force, while if an ant is in a wide stance, their middle leg would be required to compress. During upward climbing (*n*=20,314 observations across 8 collections), ants exhibited a wide stance in 79.1% of observations, while during downward climbing (*n*=18,093 observations across 8 collections), they exhibited a narrow stance in 76.6% of observations (Fig. 2B).

**Fig. 3.**
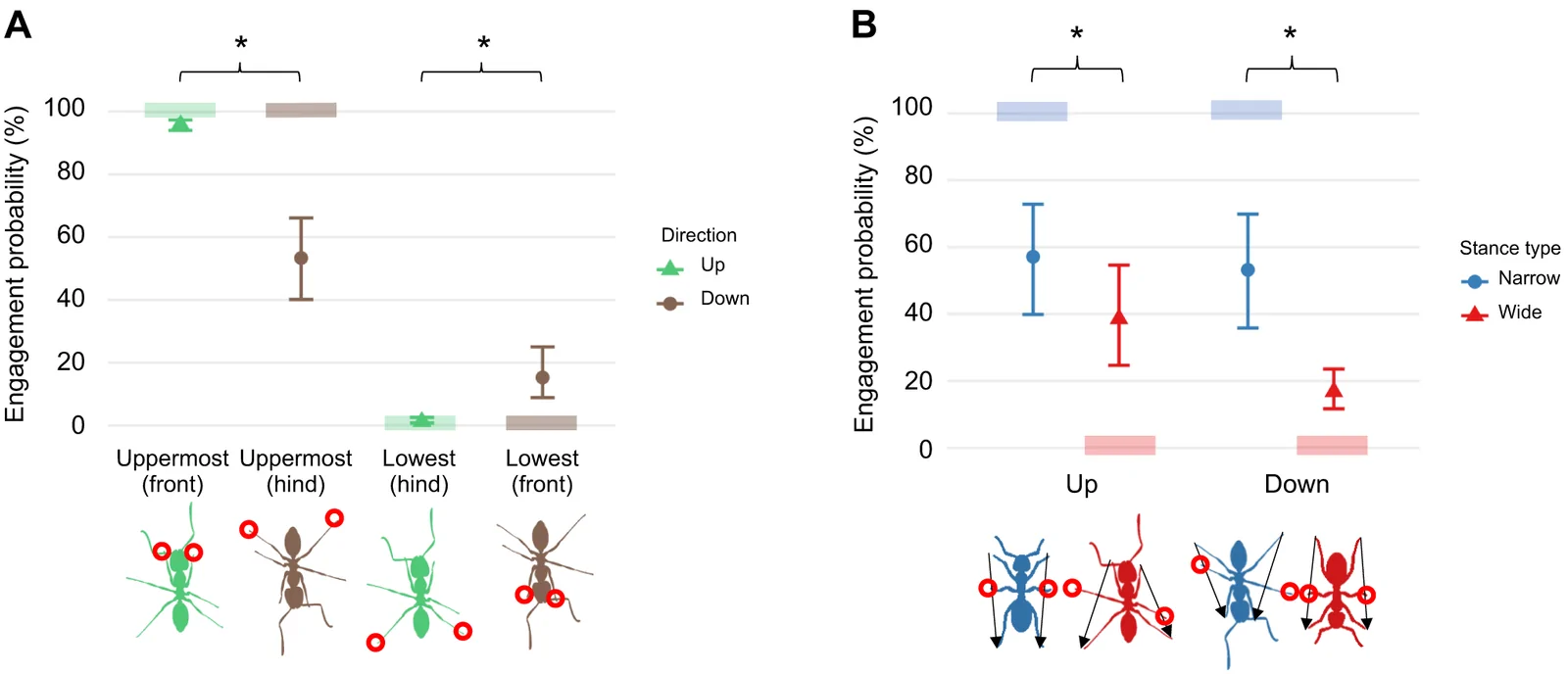
**Arolium engagement probability.** (A) Engagement probabilities for uppermost and lowest arolia. During upward climbing, the uppermost foot is the front foot and the lowest foot is the hindfoot. During downward climbing, this relationship reverses: the uppermost foot is the hindfoot and the lowest foot is the front foot. (B) Engagement probability for the middle arolium. For both A and B, points represent the pooled probability estimates across 8 collections and error bars show their 95% confidence intervals (CIs). The semi-transparent colored bars at probabilities 0% and 100% indicate the predictions from the quasi-static tripod model. The asterisks indicate significant differences.

The measured engagement probabilities of uppermost and lowest arolia showed significant differences between upward and downward climbing (Fig. 3A). During upward climbing, the uppermost (front) foot showed an arolium engagement probability of 96% (95% confidence interval, CI: [94%, 97.3%]), which was significantly higher (*z*=17.9, *P*<0.05, *k*=8) than the uppermost (hind) arolium engagement probability (53.3% [40.1%, 66.1%]) during downward climbing. Conversely, the lowest arolium had significantly lower engagement probability (*z*=−10.88, *P*<0.05, *k*=8) during upward climbing (1.2% [0.6%, 2.3%]; lowest arolium is on the hind limb) than during downward climbing (15.3% [8.9%, 25%]; lowest arolium is on the front limb). During upward climbing, the engagement status of uppermost and lowest arolia matched the required normal forces predicted by the quasi-static model in over 90% cases, while during downward climbing, less than 70% matched.

The measured engagement probabilities of the middle arolium did not differ significantly between upward (narrow: 57.1% [39.9%, 72.8%]; wide: 38.6% [24.7%, 54.6%]) and downward climbing (narrow: 53.2% [35.8%, 69.9%]; wide: 16.8% [11.7%, 23.6%]) for both stance types (narrow: *z*=0.92, Holm–Bonferroni adjusted *P*=0.36, *k*=8; wide: *z*=2.2, Holm–Bonferroni adjusted *P*=0.056, *k*=8). The middle arolium exhibited significant differences between wide stance and narrow stance when ants climbed in both directions (up: *z*=−3.08, Holm–Bonferroni adjusted *P*<0.05, *k*=8; down: *z*=−7.89, Holm–Bonferroni adjusted *P*<0.05, *k*=8; Fig. 3B). The probability that the engagement status matched the required normal forces ranged from 53.2% to 83.2% for middle arolium. Forest plots showing variances across collections are provided in Figs S5 and S6.

### Relationship between arolium engagement intensity ratio and required adhesion force ratio

**Fig. 4.**
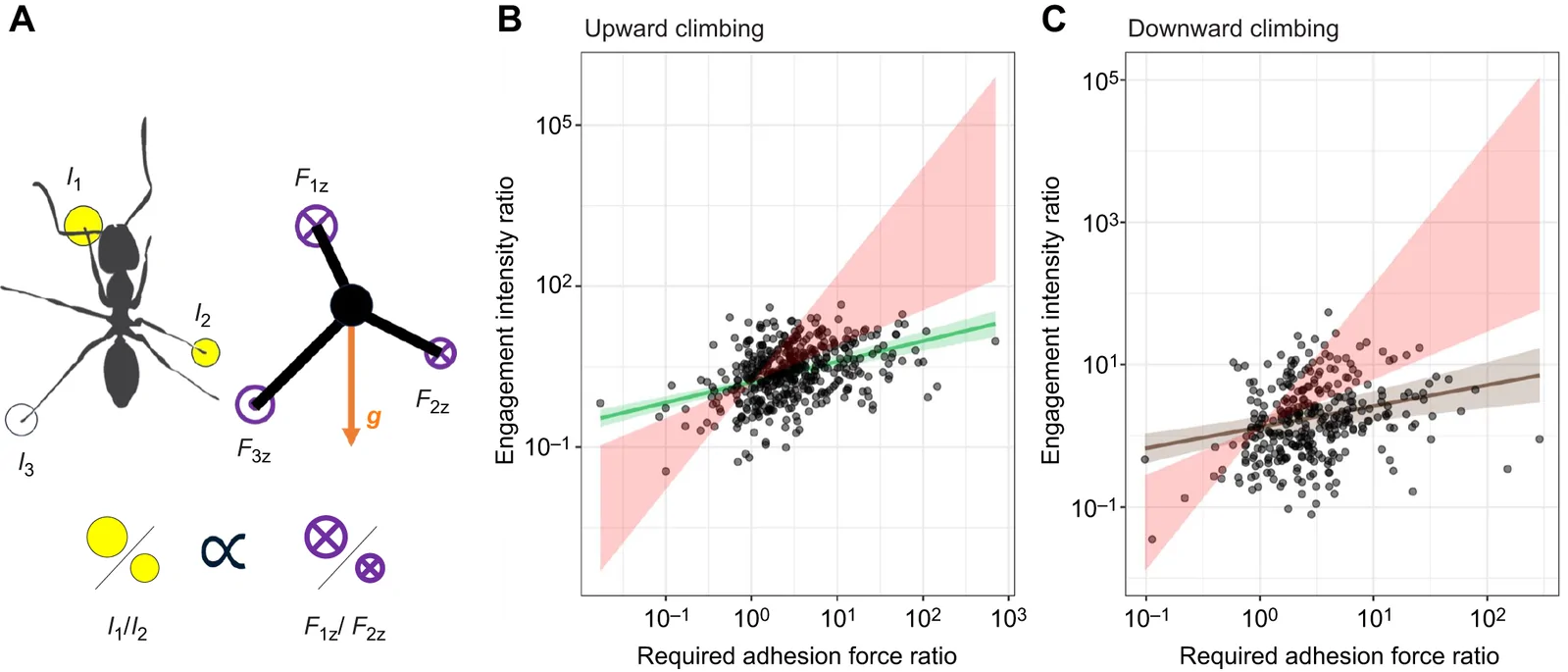
**Arolium engagement intensity ratio and required adhesion force ratio.** (A) Diagram showing the calculation of arolium engagement intensity and adhesion force ratios. Gravity (***g***) is represented by the orange arrows. Circles labeled as *I*_1,2,3_ represent arolium engagement intensity, where the white circle (*I*_3_) indicates zero intensity and the yellow circles (*I*_1_ and *I*_2_) indicate non-zero intensity. The size of each yellow circle represents the average magnitude of the arolium engagement intensity across each step. The purple symbols indicate the required force direction: ⊗ (labeled *F*_1*z*,2*z*_) denotes the required adhesion force, while ⊙ (labeled *F*_3*z*_) denotes the required compression force. The size of ⊗ represents the average magnitude of the required adhesion force across each step predicted by the tripod model. We hypothesize that the engagement intensity ratio (*I*_1_/*I*_2_) between two different arolia should be positively related to the ratio of their required adhesion forces (*F*_1*z*_/*F*_2*z*_). (B,C) Relationship between arolium engagement intensity ratio and required adhesion force ratio during climbing up (B) and climbing down (C). For both B and C, gray points are the steps across all collections (*n*=450 steps for climbing up; *n*=331 steps for climbing down). The fitted line shows the overall power-law relationship synthesized across 8 collections, with the shaded area indicating the 95% CI (green for up, brown for down). The engagement intensity ratios increased with the required adhesion force ratios for both climbing directions (meta-analysis, *P*<0.05 for both directions). The red shaded area represents the expected range of the power-law relationship between real contact area and adhesion force. Note that both axes are on logarithmic scales (log–log plot).

For steps where the quasi-static model predicted that both the uppermost and the middle feet (front and middle during upward climbing; hind and middle during downward climbing) needed to generate adhesion forces (*F*_1*z*_, *F*_2*z*_) and the observed arolium engagement intensities (*I*_1_, *I*_2_) were non-zero, we tested whether the leg requiring higher adhesion force showed more intense arolium engagement. Specifically, we analyzed the relationship between the arolium engagement intensity ratio (*I*_1_/*I*_2_) and the required adhesion force ratio (*F*_1*z*_/*F*_2*z*_) of the two limbs during upward and downward climbing (Fig. 4A). The engagement intensity ratio increased with the required adhesion force ratio for both climbing directions. During upward climbing, the engagement intensity ratio (*I*_1_/*I*_2_) scaled as (*F*_1*z*_/*F*_2*z*_)^0.38^ (95% CI: [0.30, 0.46], *z*=9.45, *P*<0.05, *k*=8; Fig. 4B), and during downward climbing, the engagement intensity ratio (*I*_1_/*I*_2_) scaled as (*F*_1*z*_/*F*_2*z*_)^0.3^ (95% CI: [0.16, 0.44], *z*=4.13, *P*<0.05, *k*=8; Fig. 4C). Although the overall scaling exponent was positive for downward climbing, individual scaling exponents for collections C0620, C0627 and C0703 were negative. Forest plots showing scaling exponents for individual collections are provided in Fig. S7.

However, the scaling exponents in both climbing directions were significantly lower than the expected range (0.67–2), as the entire 95% CIs fell below 0.67 (Fig. 4B,C). The expected range was estimated based on the pull-off adhesion forces generated by an individual arolium and by the whole animal (Labonte and Federle, 2015; Labonte et al., 2019). Adhesion stress was found to increase with shear force acting on the arolium. From a case with zero shear force to a case with shear force equal to body weight, the adhesion force (*F*_a_) generated by an arolium was expected to vary from *m*^1/3^ to *m*^1^ (where *m* is body mass). Under isometric scaling (geometric similarity), arolium area (*A*) was predicted to scale with body mass as *A*∼*m*^2/3^, so we expected the observed scaling exponent between the engagement intensity ratio (a proxy for real contact area) and the required adhesion force ratio to fall within the range 0.67–2 (*A*∼*F*_a_^0.67–2^).

### Arolium engagement with respect to time

**Fig. 5.**
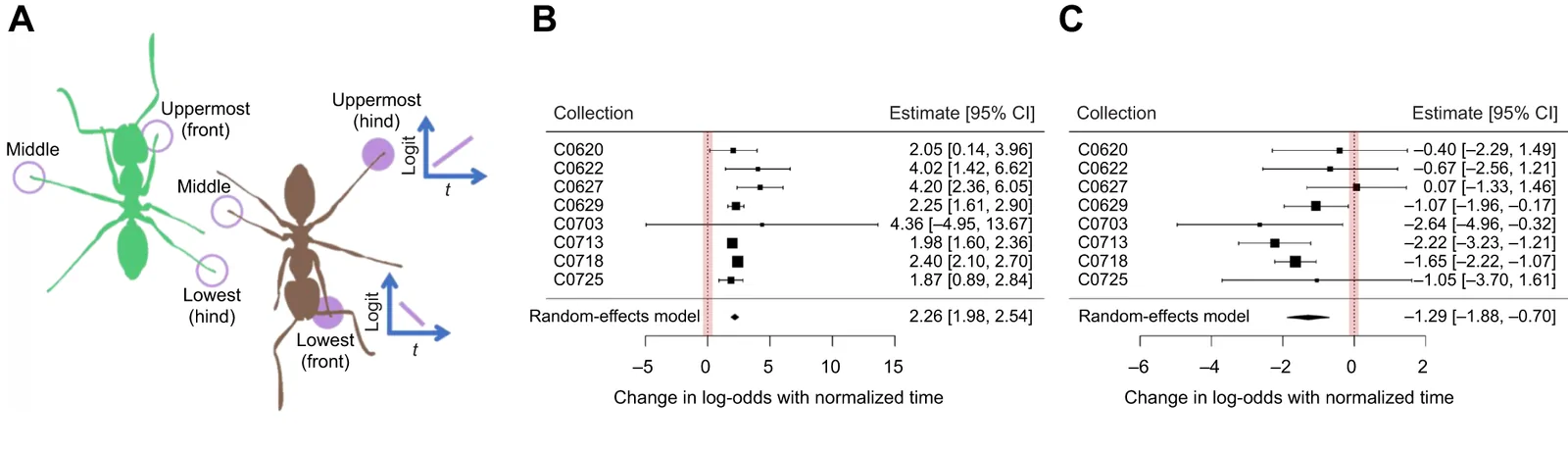
**Temporal variation of arolium engagement probability.** (A) Results overview. The ant body in green represents the climbing up scenario, while the ant body in brown represents the climbing down scenario. Feet labeled with purple circles are those in the stance phase. Among them, feet marked with solid circles showed a time-dependent engagement probability. The purple lines in the blue coordinates indicate whether the probability increases or decreases with respect to time (*t*). (B,C) Plots showing estimated slopes and 95% CIs for the relationship between the log-odds of arolium engagement and normalized time across 8 collections during climbing down for the uppermost (hind) arolium (B) and lowest (front) arolium (C). Each point represents the estimated slope from a single collection, and the horizontal lines indicate the corresponding 95% CIs. The black diamond below the collection values shows the pooled slope estimate and its 95% CI of all collections, and the vertical dashed line with red shading at slope=0 indicates no temporal variation of engagement probability. Data to the left of the vertical dashed line (negative values) indicate a decreasing trend in engagement probability, whereas data to the right (positive values) indicate an increasing trend.

We next investigated whether the engagement status of each arolium stayed the same during a stance by studying: (1) the probability of arolium engagement over time and (2) the intensity of arolium engagement over time. For engagement probability during downward climbing, temporal variation was significant for uppermost (hind arolium; *z*=15.99, Holm–Bonferroni adjusted *P*<0.05, *k*=8) and lowest arolia (front arolium; *z*=−4.29, Holm–Bonferroni adjusted *P*<0.05, *k*=8), but not for the middle arolium (narrow stance: *z*=−1.47, Holm–Bonferroni adjusted *P*=0.57, *k*=8; wide stance: *z*=0.7, Holm–Bonferroni adjusted *P*=1, *k*=8; Fig. 5A). During upward climbing, none of the arolia showed significant temporal variation in engagement probability (Holm–Bonferroni adjusted *P*>0.05 for all arolia). When Argentine ants climbed down, the arolium engagement log-odds for the uppermost arolium increased (pooled slope estimate: 2.26 [1.98, 2.54]; Fig. 5B), and the log-odds for the lowest arolium decreased (pooled slope estimate: −1.29 [−1.88, −0.7]; Fig. 5C) over the normalized time.

We then studied the arolium engagement intensity over time for adhering feet. As a stance begins and ends with no contact area, we expected the temporal pattern of engagement intensity would increase to a maximum and then decrease, following a peak-shaped second-order polynomial pattern. The middle arolium showed a relatively weaker second-order polynomial pattern: more than three collections had adjusted *R*^2^ values below 0.1, indicating substantial variance not explained by the polynomial model. For uppermost and lowest arolia, engagement intensity versus time across all collections showed a peak-shaped pattern. The only exception was the lowest (hind) arolium during upward climbing (Fig. S8). Thus, we focus the remaining analysis on the uppermost arolium during upward climbing and uppermost and lowest arolia during downward climbing.

**Fig. 6.**
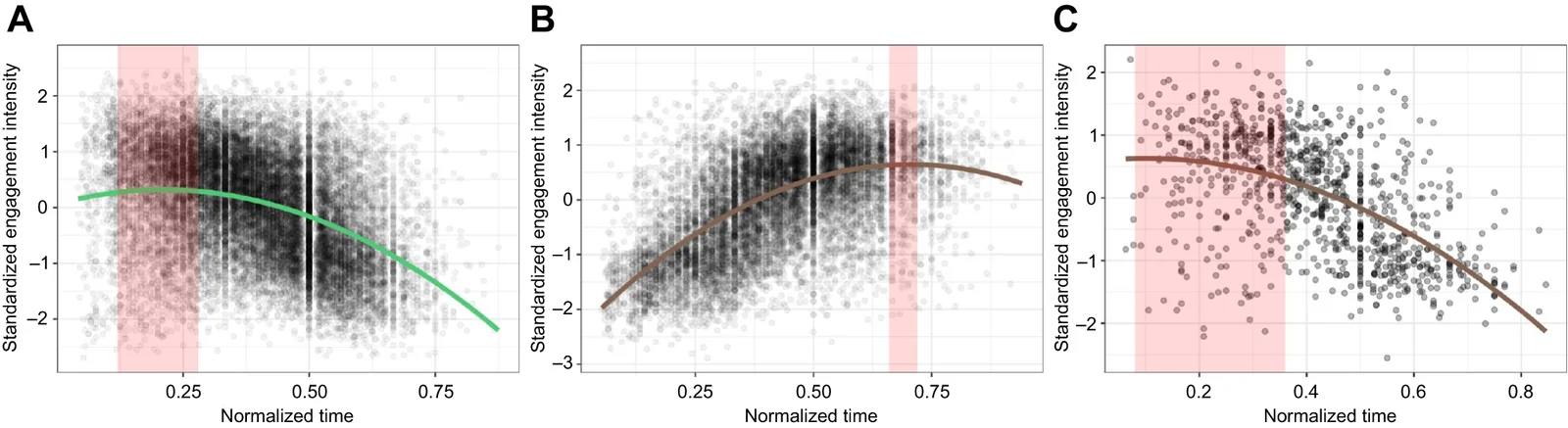
**Engagement intensity over normalized time for uppermost and lowest arolia.** (A–C) Engagement intensity for the uppermost (front) arolium during climbing up (A), uppermost (hind) arolium during climbing down (B) and lowest (front) arolium during climbing down (C) was standardized by subtracting its mean and dividing by its standard deviation within each step. Gray points are observations across all collections (A, *n*=18,974; B, *n*=11,729; C, *n*=954). The fitted curve (green for up, brown for down) is based on the pooled coefficients obtained from the meta-analysis across 8 collections. The red shaded area indicates the 95% CI for the pooled vertex. The engagement intensity vertex for the uppermost arolium was significantly earlier than mid-stance (0.5) during climbing up (meta-analysis, Holm–Bonferroni adjusted *P*<0.05; A) and was significantly later during climbing down (meta-analysis, Holm–Bonferroni adjusted *P*<0.05; B). The engagement intensity vertex for the lowest arolium was significantly earlier than mid-stance (0.5) during climbing down (meta-analysis, Holm–Bonferroni adjusted *P*<0.05; C).

Arolium engagement intensity showed an asymmetric temporal trend, indicated by the vertices (time stamp with peak engagement intensity) being significantly different from mid-stance (0.5). During upward climbing, the engagement intensity of the uppermost (front) arolium rose briefly to an early peak and then mostly decreased for the rest of the step, indicated by a vertex significantly earlier than mid-stance (−*b*/2*a*=0.2, 95% CI: [0.12, 0.28], *z*=−7.47, Holm–Bonferroni adjusted *P*<0.05, *k*=8; Fig. 6A). In contrast, during downward climbing, the arolium engagement intensity of the uppermost (hind) arolium mostly increased, with a brief decline toward the end of the step. This trend was indicated by a vertex significantly later than mid-stance (−*b*/2*a*=0.69, 95% CI: [0.66, 0.72], *z*=13, Holm–Bonferroni adjusted *P*<0.05, *k*=8; Fig. 6B). The arolium engagement intensity of the lowest (front) arolium mostly decreased after a brief incline, indicated by a vertex significantly earlier than mid-stance (−*b*/2*a*=0.22, 95% CI: [0.08, 0.36], *z*=−4, Holm–Bonferroni adjusted *P*<0.05, *k*=8; Fig. 6C). Forest plots showing vertices for individual collections are provided in Fig. S9.

## DISCUSSION

Small insects such as Argentine ants climb smooth surfaces through the use of adhesive pads on their feet called arolia. In this work, we tested whether Argentine ants distribute their arolium engagement on different legs according to the required normal forces predicted by a quasi-static model. Our results indicated that certain aspects of arolium engagement aligned with the normal force requirements. For instance, during upward climbing, the uppermost (front) arolium always engaged and the lowest (hind) arolium mostly disengaged, closely matching the quasi-static normal force requirement. In addition, middle arolium engagement status showed dependence on the stance type but not on the climbing direction. However, the discrepancies between the measured arolium engagement and the quasi-static normal force requirement were more pronounced. For example, during downward climbing, the arolium engagement pattern was not symmetric to that during upward climbing, and the match rate with normal force requirement was less than 70%. Moreover, some arolia showed temporal variation significantly different from the baseline expectation.

### Arolia engagement probability differs significantly when ants climb in different directions

Contrary to the direction-independent force requirement (uppermost arolium generates adhesion force, lowest generates compression force), climbing direction influenced arolium engagement probability. During downward climbing, the uppermost (hind) arolium engagement probability was significantly lower and the lowest (front) arolium engagement probability was significantly higher (Fig. 3A) compared with the results during upward climbing. There are multiple potential factors for this difference.

**Fig. 7.**
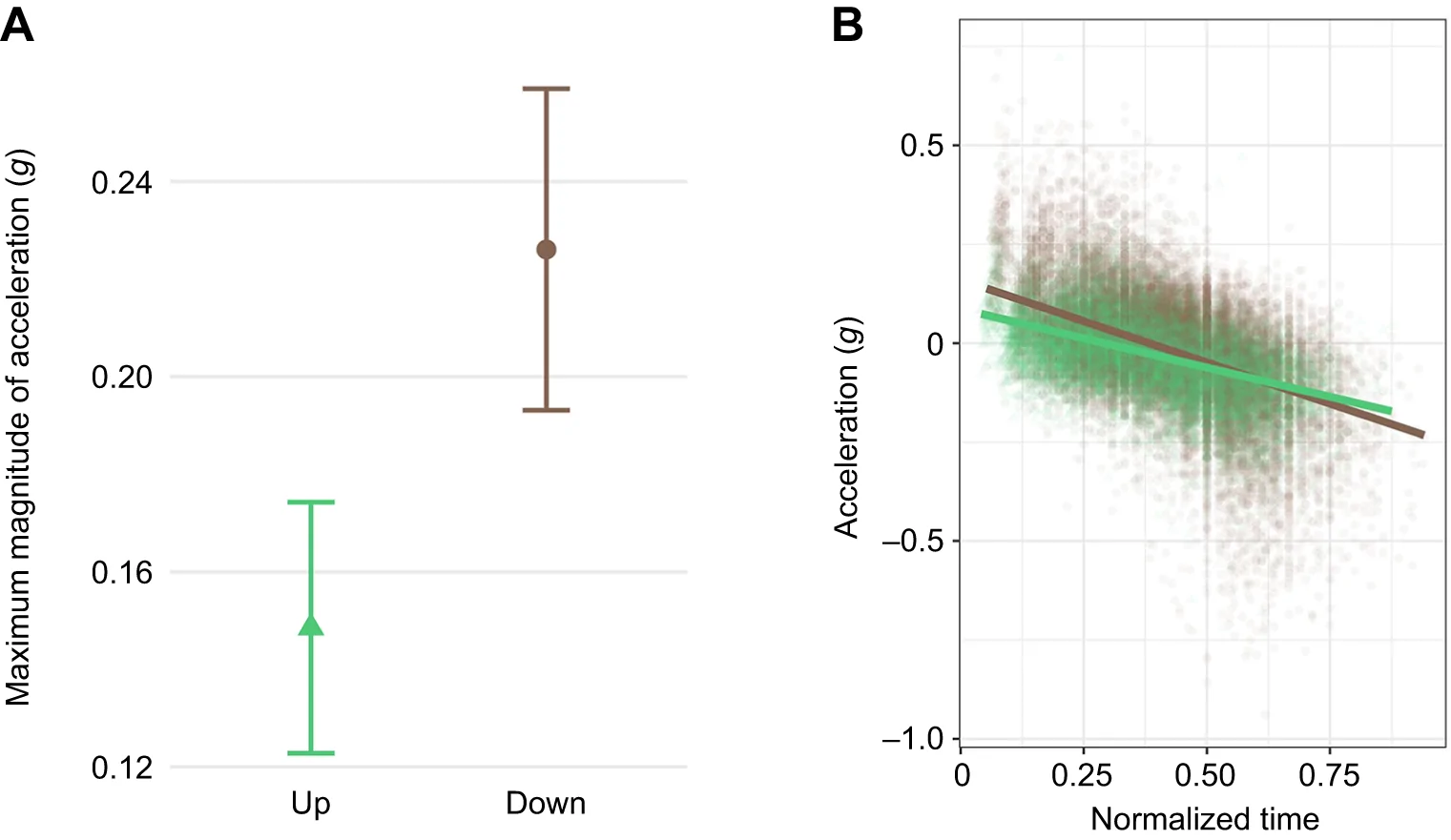
**Acceleration along the climbing direction.** (A) Maximum magnitude of acceleration along the climbing direction. Points represent the pooled estimates across 8 collections and error bars show their 95% CIs. Ants achieved significantly greater peak acceleration magnitudes during downward climbing than during upward climbing (meta-analysis, *P*<0.05). (B) Temporal variation of acceleration along the climbing direction. Green points are the climbing upward data across all collections (*n*=20,746 observations) and the green fitted line is based on the pooled intercept and slope obtained from the meta-analysis across 8 collections. The brown line (*k=*8 collections) and brown points (*n*=18,272 observations) represent the results for climbing down. *g* is 9.8 m s^−2^. During each step, Argentine ants accelerated first and then decelerated during both upward and downward climbing (meta-analysis, *P*<0.05 for both directions).

The first potential factor is the difference in body acceleration when Argentine ants climbed in different directions. Ants achieved significantly greater peak acceleration magnitude (*z*=10.61, *P*<0.05, *k*=8; Fig. 7A) during downward climbing (0.23 *g* [0.19, 0.26 *g*]) than during upward climbing (0.15 *g* [0.12, 0.17 *g*]). Higher acceleration may make downward climbing less predictable by our quasi-static model compared with upward climbing and can potentially cause greater variance in engagement probability as seen in Fig. 3A.

Furthermore, the non-isotropic body mass distribution may contribute to the observed engagement probability differences between upward and downward climbing. The quasi-static model simplified the ant body to a mass point, thereby ignoring the uneven distribution of body mass in Argentine ants. Changing mass distribution can alter limb forces, as a previous study in trotting dogs showed that changing load distribution altered individual limb mechanics and contact timing (Lee et al., 2004). This suggests that the model's simplification may partly explain why the required normal force is direction independent, while the engagement probability is not.

Lastly, we assumed foot-only substrate contact in our study. However, studies on Argentine ant trailing behavior have shown that other body parts can contact the substrate (Robertson et al., 1980; Aron et al., 1989; Reid et al., 2012). For example, when ants walked upside-down on a glass slide, their gasters were observed touching the glass (Reid et al., 2012). When ants walked on an inclined tube, pheromones on the substrate were deposited from the gaster, and ants were observed touching the substrate with their antennae (Robertson et al., 1980). Therefore, other body parts may occasionally contact the substrate during vertical climbing. If antennae, mandibles or the gaster contact the substrate and influence climbing differently depending on direction, such contact could potentially contribute to direction-dependent arolium engagement.

### Middle arolium exhibits intermediate engagement probability with high variance

Consistent with the quasi-static normal force requirement, the engagement probability of the middle arolium was direction independent but varied with stance type. However, unlike the required normal forces, its engagement probability was never close to 100% for a narrow stance case or near 0% for a wide stance case and its engagement probability always varied over a wide range.

The intermediate and inconsistent engagement of the middle arolium may result from a combination of its low normal force requirement and the absence of a consistent force pulling toward or pushing against the body. The low normal force requirement can be derived from the quasi-static model. Within a stance of the alternating tripod gait, the lateral distance between the uppermost and the lowest feet is often shorter than that between the middle and the lowest feet, which, according to the quasi-static model, results in a lower required adhesion force for the middle arolium than for the uppermost arolium.

Previous studies have shown that the extension of an arolium can be controlled passively and the passive mechanism is direction dependent (Federle et al., 2001, 2002; Endlein and Federle, 2013). The arolium extends if the corresponding leg is pulled toward the body, while the arolium folds when the leg is pushed against the body. During vertical climbing, compared with the front leg or the hindleg, the middle leg is typically oriented more perpendicularly to the direction of gravity, resulting in reduced passive shear force caused by gravity along its axis.

Furthermore, lateral body oscillation was observed in our climbing ants. Such oscillation may require pulling or pushing forces along the middle leg's direction, as reported in a previous study on wood ants running (Reinhardt et al., 2009). As the required normal force and the consistent pulling or pushing force caused by gravity are small, the inconsistent pushing and pulling forces required by lateral body oscillation may dominate the engagement status of the middle arolium. Additionally, a low required adhesion force necessitates a small engagement intensity, which may be difficult to detect accurately.

### Association between engagement intensity and required adhesion force is weak and deviates from the expected range

When it comes to the overall relationship between the engagement intensity ratio and the required adhesion force ratio, the scaling exponents were significantly positive for both climbing directions. However, some collections during downward climbing had negative scaling exponents. In addition, the data points were widely scattered around the fitted relationship, suggesting a large portion of the total variance in engagement intensity ratio remained unexplained.

One potential reason for this is that, during free climbing, ants engage their arolia across a broad range of contact areas that are all sufficient to meet both adhesion force and speed demands. This points to a functional plateau rather than a finely tuned optimum. Supporting this idea, ants exhibited high acceleration/deceleration during some fast steps (Fig. S10). This likely requires both increased adhesive force to prevent detachment and short step duration to maintain fast speed. If ants can achieve both strong adhesion force and rapid movement under such conditions, during slower steps – when both adhesion force and speed demands are lower – a range of intermediate contact areas would be sufficient. Thus, instead of optimizing contact area according to required adhesion force for every step, ants may rely on a robust adhesive system that performs effectively across a range of engagement levels. A previous study on geckos also found the same phenomenon, as they revealed setae might make contact without generating a significant normal force (Eason et al., 2015).

In addition to the large amount of unexplained variance, the measured correlation parameter was out of the expected adhesion range. This may result from a non-linear relationship between light flux and real contact area. Although the light flux increases with real contact area in FTIR studies, a previous study with a cylindrical puck pressed against an FTIR sensor suggests that deformation of the contact material can alter scattering intensity (Sharp et al., 2018).

### Arolia exhibit time-dependent engagement probability and intensity during each step

The quasi-static model predicts that throughout each climbing step, ants' foot normal forces do not change over time, even as their CoM moves relative to the supporting legs. Contrary to the predicted time independence of the required adhesion force, in our experiment we observed that the uppermost and lowest arolia during downward climbing exhibited time-dependent engagement probabilities. In addition, the uppermost arolium when climbing in both directions and lowest arolium during downward climbing showed significant asymmetric temporal changes in engagement intensity.

Time dependence of engagement intensity may come from the inherent cyclic nature of a stance (initiate engagement, adhere, disengage) or be related to locomotion mode. To assess the source of this time dependence, we compared the temporal changes in engagement intensity with previously published data on temporal variation in contact area of Asian weaver ants (*Oecophylla smaragdina*) during upside-down walking (Federle and Endlein, 2004; Fig. S11). The vertices (−*b*/2*a*) for the upside-down data were much closer to mid-stance (0.51 for middle arolium and 0.56 for hind arolium), suggesting more symmetrical engagement patterns compared with the trends we measured during climbing. These comparisons supported that during climbing, the asymmetric temporal variations in engagement intensity were not because of the cyclical nature of the engagement and disengagement processes but because of non-inherent changes related to climbing.

Among cases with time-dependent engagement, the uppermost arolium during downward climbing displayed an increasing trend in both engagement probability and intensity, whereas all other cases showed decreasing trends. One reason to explain the time-dependent engagement for the uppermost and lowest arolia may be the non-negligible body acceleration during climbing.

During each step, Argentine ants accelerated first and then decelerated (Fig. 7B; up: pooled slope=−0.3 [−0.39, −0.2], *z*=−6.15, *P*<0.05, *k*=8; down: pooled slope=−0.42 [−0.56, −0.28], *z*=−5.94, *P*<0.05, *k*=8). This temporal pattern of acceleration aligned with the temporal trend of the uppermost arolium engagement for both upward and downward climbing (Figs 5 and 6). In the static case, the normal force requirement (Eqns 3–6) is influenced by gravity alone. However, during locomotion, the body experiences both gravitational force (*mg*) and inertial forces due to acceleration (*ma*). During upward climbing, the ant's body first experiences a net upward force to overcome gravity and accelerate, then a net downward force as gravity assists in deceleration. The normal force requirement must therefore adjust to reflect this changing net load, necessitating the adhesion force on the top arolium to be stronger at the start and then decrease, aligning with the arolium engagement intensity trend in Fig. 6A. Conversely, during downward climbing, ants use gravity to accelerate initially and later need more upward force to decelerate, causing the adhesion force on the top arolium to be weaker at first and increase over time, matching the arolium engagement trend in Figs 5B and 6B.

This acceleration trend during downward climbing also requires the lowest arolium to gradually increase the compression force. As ants often do not adhere their arolium when the corresponding leg presses onto the surface (Endlein and Federle, 2015), we expected the lowest arolium engagement intensity to change in the opposite direction to the compression force requirement. The measured decreasing trend (Figs 5C and 6C) matched our expectation. Thus, we hypothesize that body accelerations are important in ant climbing dynamics, which may be one explanation for why the quasi-static force requirement fails to predict the observed temporal engagement trends.

Another possible explanation is that the distance between the ant's CoM and the contacting surface may vary over time. Previous studies on level walking in *Formica pratensis*, *Cataglyphis fortis* (Weihmann and Blickhan, 2009) and *Formica polyctena* (Reinhardt and Blickhan, 2014) and slope traversing in *F. pratensis* and *C. fortis* (Weihmann and Blickhan, 2009) reported small body height oscillations, with height variance below 10% of body height in most cases, supporting the validity of our constant CoM-to-substrate distance assumption in calculating normal force requirement. However, it is unknown whether CoM-to-substrate distance oscillations during vertical climbing remain similarly small. Therefore, future studies should incorporate 3D body kinematics to account for varying CoM-to-substrate distance when calculating the normal force requirement.

### Comparison with previous studies suggests some findings may apply beyond Argentine ants

Consistent with the asymmetric arolium engagement pattern, a study on *C. fortis* climbing slopes reported asymmetric leg impulses between ascending and descending. When *C. fortis* walked on a 60 deg upslope, the front (uppermost) legs mostly generated compression impulses in the normal direction, while on a 60 deg downslope, the hind (uppermost) legs mostly generated adhesion impulse (Wöhrl et al., 2017).

During level walking, *F. polyctena* displayed acceleration ranging from approximately −2 to 1.5 m s^−2^ within a single step (Reinhardt and Blickhan, 2014), which matches the magnitude we measured in Argentine ants during vertical climbing (up: from −1.69 to 0.72 m s^−2^; down: from −2.28 to 1.35 m s^−2^). This agreement suggests that arolium engagement in other ant species may also be impacted by within-step acceleration.

Moreover, such non-negligible within-step acceleration was not only observed in ants but also in other insects. During rapid vertical climbing, cockroaches showed fore–aft forces oscillating around body weight. The peak fore–aft acceleratory forces generated by cockroaches were around 1.7 times body weight, while the minimum values were around 0.26 times body weight (Goldman et al., 2006), indicating an acceleration range from −6.27 to 6.86 m s^−2^ within a stride. Based on figure 4 from Chun et al. (2021), within a fast step, the speed change of *Drosophila* could reach up to around 60% of its average speed, indicating a non-negligible acceleration.

### Conclusion

In conclusion, the measured arolium engagement of Argentine ants showed an asymmetric pattern between upward and downward climbing, and changed over time within a single step. These characteristics could not be explained by the normal force requirement of a quasi-static model during vertical climbing. This disagreement may arise from several possible factors including: (1) non-negligible within-step body accelerations; (2) anatomical complexity, including unevenly distributed body mass that violates the simplified point-mass assumption of the model; (3) passive mechanical coupling between leg forces and arolium extension; and (4) the ants' flexibility to select contact areas within a broad range that appears sufficient to meet both adhesion and speed demands. Future studies incorporating simultaneous force measurements across feet may help determine the arrangement and control of arolium engagement in rapid climbing.

## Supplementary Material

10.1242/jexbio.253096_sup1 Supplementary information
